# Risk Factors for Internal Jugular Vein Thrombosis 1 Month After Non-Cuffed Hemodialysis Catheter Removal

**DOI:** 10.3390/jcm13247579

**Published:** 2024-12-13

**Authors:** Shun Yoshida, Yasuyo Sato, Tsukasa Naganuma, Ikuo Nukui, Masakiyo Wakasugi, Ayumu Nakashima

**Affiliations:** 1 Department of Nephrology, Yamanashi Prefectural Central Hospital, Yamanashi 400-8506, Japan; oyasuyo@yamanashi.ac.jp (Y.S.); naganuma-bfpn@ych.pref.yamanashi.jp (T.N.); nukui-akfg@ych.pref.yamanashi.jp (I.N.); m-wakasugi@ych.pref.yamanashi.jp (M.W.); 2 Department of Nephrology, University of Yamanashi Hospital, Yamanashi 400-8506, Japan; a.nakashima@yamanashi.ac.jp

**Keywords:** temporary catheter, non-tunneled catheter, lymphadenopathy

## Abstract

**Background**: Complications, namely, catheter-related thrombosis (CRT) and venous stenosis, are associated with non-cuffed hemodialysis catheters used for emergency vascular access. However, only a few reports have demonstrated changes in the venous lumen and intravenous thrombosis after catheter removal. In this study, we comprehensively investigated the risk factors for residual thrombus 1 month after hemodialysis catheter removal. **Methods**: This prospective observational study was conducted from June 2021 to October 2022. We included patients with end-stage kidney disease who underwent hemodialysis catheter placement in the internal jugular vein (IJV). After catheter removal, we observed the IJV using vascular ultrasound and evaluated the thrombus and vein properties. Furthermore, we observed thrombosis 1 month after catheter removal, and investigated the risk factors for residual thrombus 1 month after catheter removal. **Results**: A thrombus was observed at the site of catheter removal in all the cases. Of the 37 patients who were followed up, 11 exhibited a residual thrombus 1 month after catheter removal. Patients with arteriovenous (AV) access dysfunction and enlarged lymph nodes during catheter removal were significantly more likely to have a residual thrombus 1 month after catheter removal. These associations remained significant even after adjusting for age, sex, and diabetes status. **Conclusions**: In 29.7% of the patients, CRT persisted even 1 month after the removal of the non-cuffed hemodialysis catheter. The provision of early intervention in patients with AV access dysfunction and enlarged lymph nodes during catheter removal may prevent CRT persistence.

## 1. Introduction

Vascular access is essential for blood purification therapies such as hemodialysis. Non-cuffed hemodialysis catheters are vascular accesses used for urgency and short-term practice [1]. Complications after catheter placement include catheter dysfunction, catheter-related bloodstream infections (CRBSIs), catheter-related thrombosis (CRT), and central venous (CV) stenosis [2,3,4]. Hemodialysis catheters are inserted in the same way as general CV catheters and are managed in the same way. However, hemodialysis catheters have larger diameters than CV catheters for blood purification therapy. Therefore, complications are more frequent after catheter placement [5]. Clinicians always need to pay close attention to the occurrence of complications.

McFall et al. reported that CV stenosis occurs in 20% to 40% of the cases owing to indwelling hemodialysis catheters [6]. Wang et al. reported that the relationship between CV stenosis development and thrombosis remains unclear; nonetheless, it is associated with a history of CV placement [7]. Thapa et al. reported on a case of CRT combined with superior vena cava (SVC) syndrome and pulmonary embolism (PE) [8]. We have reported on two cases of internal jugular vein (IJV) stenosis after aseptic thrombophlebitis development associated with non-cuffed hemodialysis catheters [9].

The frequency of CRT after catheter removal ranges from 25.9% to 58.3% [10,11,12]. Few researchers have investigated the rate of residual thrombus after catheter removal and the influencing risk factors. In this study, we investigated the factors related to CRT 1 month after hemodialysis catheter removal in patients with recent hemodialysis or arteriovenous (AV) access dysfunction who could complete an ultrasonographic evaluation up to 1 month after catheter removal.

## 2. Materials and Methods

### 2.1. Study Population

This single-center prospective observational study was conducted at the Yamanashi Prefectural Central Hospital. Patients aged ≥ 20 years with renal failure and with a hemodialysis catheter (non-cuffed catheter) inserted into the IJV were included. The patients were newly introduced to hemodialysis or presented with AV access dysfunction. This study was performed following the tenets of the Declaration of Helsinki. The study protocol was approved by the Ethical Review Committee of Yamanashi Prefectural Central Hospital (Clinical 2021-6, 1 June 2021), and written consent was obtained from all the patients.

### 2.2. Data Collection

**Figure 1:**
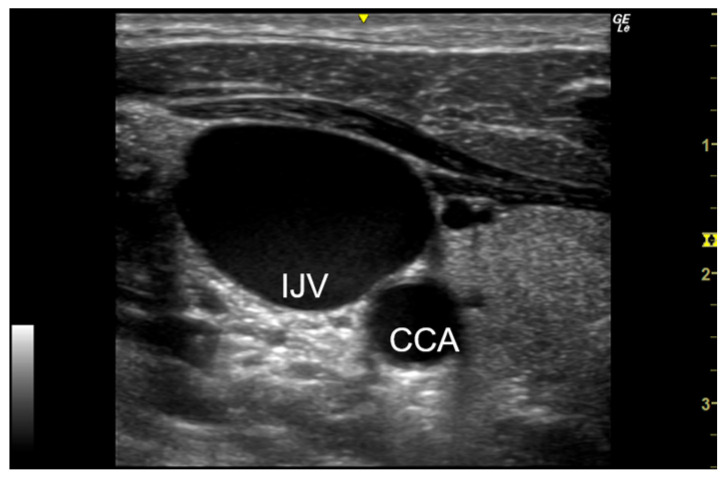
Ultrasonographic images of a normal IJV (transverse section). IJV, internal jugular vein; CCA, common carotid artery.

**Figure 2:**
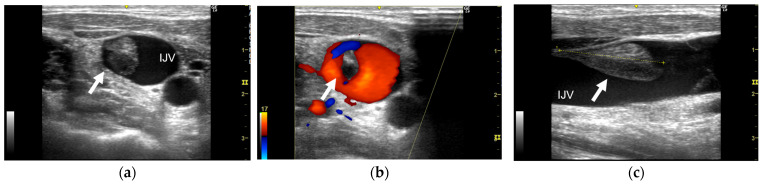
Ultrasonographic images of an IJV thrombus (arrows) confirmed after catheter removal. (**a**): transverse section; (**b**): color Doppler imaging; (**c**): longitudinal section. Color Doppler imaging shows blood flow outside the thrombus. IJV, internal jugular vein.

We collected patient information (age, sex, underlying disease, reason for hospitalization, complications, medications, and previous catheterization) and blood test data during catheter placement and removal to investigate the factors related to CRT. We evaluated intravenous thrombus formation, venous changes around the insertion site (venous lumen diameter, stenosis, and occlusion while holding breath after inspiration), and surrounding enlarged lymph nodes using the LOGIQ e Premium (GE HealthCare, Chicago, IL, USA) vascular ultrasound device. Figure 1 shows the normal findings of the IJV and Figure 2 shows the ultrasonographic findings of the IJV thrombus. As a rule, we observed the thrombus in transverse and longitudinal sections on ultrasonography (US).

**Figure 3:**
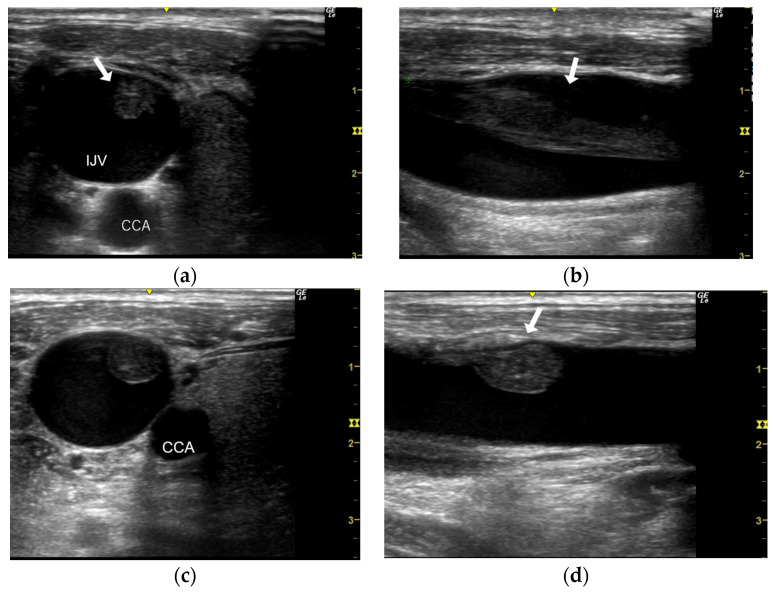

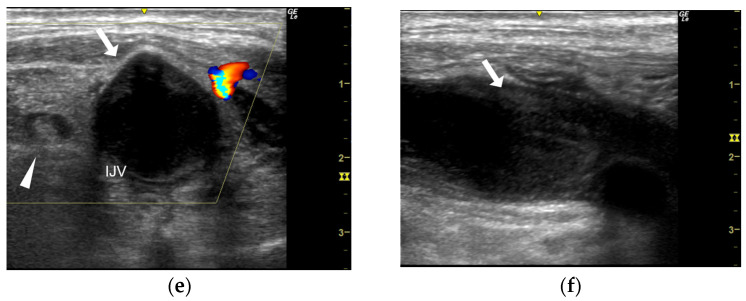
Various thrombus shapes (arrows). (**a**,**b**) Fibrin sheath (FS) is a thrombus that appears as a cord-like structure. (**c**,**d**) Images show FS with mural thrombus. (**e**,**f**) Images show thrombus filling and occluding the IJV. Color Doppler images show no blood flow in the IJV. An enlarged lymph node (arrowhead) near the obstructed IJV was also observed. IJV, internal jugular vein; CCA, common carotid artery.

AV access dysfunction is defined as a condition in which arteriovenous fistulas (AVFs) or arteriovenous grafts (AVGs), which serve as vascular access devices for hemodialysis, become compromised due to vascular stenosis, thrombosis, infection, or other complications [13]. Thrombus resolution was defined as the absence of thrombus on cross and longitudinal images. The thrombus was defined as a fibrin sheath (FS)-type when it was observed as a cord-like structure extending from the catheter insertion site (Figure 3a,b). And, FS with mural thrombus was classified as a mixed thrombus/FS type (Figure 3c,d). Complete thrombus occlusion (CTO) was defined as the obstruction of the internal jugular vein by a thrombus (Figure 3e,f). Enlarged lymph nodes were defined as the presence of one or more new lymph nodes with a short diameter of 5 mm or greater around the catheter insertion site at the time of catheter removal (Figure 3e).

These observations were recorded before catheter insertion, immediately before and after catheter removal, on days 1 and 3 (±1 d), and at 1 month (day 28 ± 4 days) after catheter removal. The follow-up was terminated if the thrombus was confirmed to have disappeared before 1 month after catheter removal. If the thrombus persisted 1 month after catheter removal, ultrasonography was continued until it disappeared completely. Furthermore, the symptoms at the time of catheter removal were assessed.

### 2.3. Statistical Analysis

Data are presented as mean values ± standard deviation (SD) or median and interquartile range (25th–75th percentiles). The Mann–Whitney U test or χ^2^ test was conducted for comparison between the groups. Additionally, logistic regression was used to assess the independent predictors of CRT 1 month after catheter removal. *p* < 0.05 was considered statistically significant.

## 3. Results

### 3.1. Patient Characteristics

Between June 2021 and October 2022, 44 patients with acute or chronic renal failure underwent hemodialysis catheter placement in the IJV. Thirty-seven patients completed ultrasonographic evaluation within 1 month of catheter removal. The remaining patients were lost to follow-up. Three patients each were transferred and underwent catheter reinsertion; one died (Figure S1). Table 1 presents the clinical characteristics of 37 patients. The mean age was 76 years (69.0–83.0 years), and 25 (67.6%) were men. Eleven (29.7%) patients exhibited residual thrombi 1 month after catheter removal. The reasons for admission were dialysis initiation and AV access dysfunction in 29 (78.4%) and 8 (21.6%) patients, respectively. Five (13.5%) patients experienced acute renal failure. Eighteen (48.6%) patients had diabetes mellitus (DM), and eight (21.6%) underwent previous CV catheter insertion at an identical site. The median duration of catheter placement was 17.0 days. There were two cases of bacteremia with suspected CRBSI at catheter removal (Table 2). Twelve (32.4%) patients were symptomatic at the time of catheter removal. Fever was the most common symptom (eight patients), followed by sore throat (four patients) and headache (one patient). Thrombophlebitis was observed in one patient, but there were no complications, such as PE or venous hypertension. No symptoms or complications (bleeding, subcutaneous hematoma, or air embolization) were associated with catheter removal. The use of anticoagulants was limited to dialysis sessions only in all the cases. No patient received continuous heparin administration during the catheter placement period.

### 3.2. Factors Associated with CRT 1 Month After Hemodialysis Catheter Removal

We evaluated 37 patients up to 1 month after catheter removal and classified them into thrombus and non-thrombus groups. The rate of AV access dysfunction was significantly higher in the thrombus group than in the non-thrombus group (*p* = 0.04). We observed no significant differences in the age, sex, DM, medications (iron, anti-thrombotic agents, erythropoiesis-stimulating agents, and hypoxia-inducible factor-prolyl hydroxylase inhibitors) or previous CV catheter placement between the groups (Table 1).

The thrombus group demonstrated a significantly higher rate of enlarged lymph nodes at the time of catheter removal (*p* = 0.04). Additionally, the thrombus during catheter removal was classified into FS type, mixed thrombus/FS type, and CTO type. All four patients (100%) exhibited residual thrombi even 1 month after catheter removal in the CTO type. By contrast, none of the five patients exhibited a thrombus 1 month after catheter removal in the mixed thrombus/FS type. We observed no significant differences in the catheter diameter, indwelling catheter days, bacteremia, or symptoms between the groups (Table 2). The rate of decline in hemoglobin (Hb) was significantly higher in the thrombus group (−9.4% [−25.4 to 2.9] vs. 3.0% [−10.6 to 9.9], *p* = 0.04) (Table 3).

AV access dysfunction (odds ratio: OR 6.39; 95% confidence interval: CI 1.18 to 34.62; *p* = 0.03), enlarged lymph nodes (OR 6.39; 95% CI 1.18 to 34.62; *p* = 0.03), and the rate of decline in Hb (Per 1%, OR 0.95; 95% CI 0.90 to 1.00; *p* = 0.04) were significantly higher in the thrombus group than in the non-thrombus group. Next, we investigated whether these significant differences remained even after adjusting for the background of patients such as age, sex, and DM. AV access dysfunction and enlarged lymph nodes were the independent risk factors for residual thrombus even after adjusting for age, sex, and DM. In contrast, the difference in the rate of Hb level decline disappeared after adjusting for age, sex, and DM (Table 4).

Nine patients in the thrombus group were observed for up to 3 months after catheter removal, and three (33.3%) had residual thrombi.

## 4. Discussion

CRT caused by non-cuffed hemodialysis catheter placement persisted in 29.7% of the patients 1 month after catheter removal. CRT is more likely to persist in patients with AV access dysfunction than in patients who have recently started dialysis. Additionally, patients with completely occluded CRT during catheter removal and patients with enlarged lymph nodes were more likely to have a residual thrombus even after 1 month of catheter removal. Thus, early intervention in patients with AV access dysfunction and enlarged lymph nodes during catheter removal may prevent persistent CRT.

Our results suggest that CRT is more likely to occur in patients with AV access dysfunction. Venous stenosis associated with intimal thickening is the primary cause of AV access dysfunction; however, thrombotic risk factors, such as genetic polymorphisms (including methylenetetrahydrofolate reductase), coagulation factors (factor XIII, prothrombin, and factor V Leiden), and neutrophil extracellular traps, are also associated with AV access dysfunction [14,15,16]. Therefore, patients with AV access dysfunction may have thrombotic risk factors that supposedly influence the residual thrombus. Taken together, patients with AV access dysfunction should be evaluated using ultrasonography after catheter removal for thrombus status and assessed for the development of CV stenosis, venous hypertension, or SVC syndrome. The indications for catheter insertion should be evaluated carefully in patients with AV access dysfunction. To avoid unnecessary catheter insertion, clinicians should focus on daily AV access management.

Wilkin et al. reported on CRT in 25.9% of the patients with indwelling hemodialysis catheters; however, this proportion differs from our finding in that it was based on patients with cuffed catheters and included silicone catheters [10]. Oguzkurt et al. reported on a 56.0% fibrin sheath during non-cuffed hemodialysis catheter placement in patients under maintenance hemodialysis [11]. Yardim et al. reported on a significant association between CRT and previous catheter insertion in a prospective observational study, with 58.3% of the CRT observed immediately after hemodialysis catheter removal and a mean indwelling duration of 55 days [12]. In our study, CRT was confirmed in all the patients immediately after catheter removal. Our results differ from the previous findings, which may be attributed to the use of a non-cuffed hemodialysis catheter. In this study, we observed no significant association of residual thrombus 1 month after catheter removal with the duration of catheter placement or a history of catheter insertion. Catheter diameter and CRBSI development are the risk factors for CRT [3]; however, none were significantly associated with residual thrombus 1 month after catheter removal in this study. In contrast, enlarged lymph nodes were the independent risk factors for residual thrombus, despite no reports on an association between CRT and enlarged lymph nodes. Enlarged lymph nodes suggested local inflammation that occurred with catheter placement. The presence of inflammation around the site of catheter placement may be associated with the prolonged persistence of thrombus.

In this study, the residual thrombus was also prevalent in CTO types. This can be attributed to several factors: larger thrombi require more time for spontaneous resolution, the stasis of blood flow leads to slower fibrinolytic activity, and the thrombus itself can induce local inflammation of the venous wall.

The prophylactic use of anticoagulants for CRT is not recommended, and the optimal treatment strategy remains uncertain [17,18]. In this study, all the patients developed CRT, which resolved spontaneously without follow-up treatment and did not result in any serious complications such as PE. Conversely, some patients exhibited long-term residual thrombus. Further studies are required to determine if anticoagulation therapy is effective in preventing complications secondary to CRT.

In recent years, efforts to develop device materials with enhanced anti-thrombotic and anti-inflammatory properties have been ongoing [19,20]. And, factor (f) XII activation has been implicated as a cause of device-induced thrombosis, including CV catheters [20]. Molecular agents targeting fXII and fXI have been reported to inhibit thrombosis associated with intravascular devices in animal models [21,22,23,24]. fXII and fXI have the advantage of being less involved in the hemostatic mechanism, which suggests a high safety profile. These agents, which are anticipated to be developed in the future, may not need to be indicated for all patients requiring medical devices, but rather for a narrowly targeted patient population. Our results could influence the selection of patients who require more intensive thromboprophylaxis.

A limitation of this study was the small sample size. This drawback may have prevented a better selection of the factors affecting residual thrombus 1 month after catheter removal, thus warranting multicenter prospective studies in the future.

## 5. Conclusions

Patients with AV access dysfunction and enlarged lymph nodes at the time of catheter removal were more likely to have residual thrombi 1 month after catheter removal. We recommend follow-up of the thrombus after catheter removal in such patients.

## Figures and Tables

**Table 1:** Baseline characteristics and parameters at catheter insertion.

	All (n = 37)	Thrombus Group (n = 11)	Non-Thrombus Group (n = 26)	*p*-Value
Age, years	76 (69 to 83)	75 (61 to 77)	79 (70 to 83)	0.10
Male, n (%)	25 (67.6)	8 (72.7)	17 (65.4)	0.66
^a^ AV access dysfunction, n (%)	8 (21.6)	5 (45.5)	3 (11.5)	0.04
DM, n (%)	18 (48.6)	4 (36.4)	14 (53.8)	0.28
Acute kidney injury, n (%)	5 (13.5)	1 (9.1)	4 (15.4)	0.61
Use of iron preparations, n (%)	8 (21.6)	3 (27.3)	5 (19.2)	0.30
Treatment of renal anemia (None:ESA:HIF-PHI)	6:25:6	3:5:3	3:20:3	0.17
Previous history of catheterization, n (%)	8 (21.6)	3 (27.3)	5 (19.2)	0.59
WBC (/μL)	5900 (4600 to 8000)	5900 (5350 to 8100)	5900 (4375 to 6975)	0.65
Hb (g/dL)	9.3 (8.4 to 11.2)	11.4 (8.9 to 12.5)	9.0 (8.3 to 10.2)	0.07
Plt (×10^5^/μL)	15.4 (11.2 to 20.3)	18.9 (14.9 to 23.2)	13.9 (8.8 to 19.0)	0.09
Fib (mg/dL)	385 (332 to 453)	397 (383 to 425)	360 (326 to 474)	0.44
D-dimer (μg/mL)	4.6 (2.3 to 9.3)	3.3 (1.9 to 10.8)	5.1 (2.9 to 8.9)	0.71
T-SAT (%)	25.3 (19.4 to 50.0)	28.6 (22.6 to 47.0)	20.9 (15.7 to 51.4)	0.50
Ferritin (ng/mL)	169.6 (66.9 to 382.1)	153.7 (52.7 to 195.0)	261.9 (67.1 to 512.1)	0.30
CRP (mg/dL)	1.0 (0.2 to 5.5)	1.0 (0.3 to 3.6)	1.0 (0.2 to 7.7)	0.86

^a^ AV, arteriovenous; DM, diabetes mellitus; ESA, erythropoiesis-stimulating agent; HIF-PHI, hypoxia-inducible factor-prolyl hydroxylase inhibitor; Hb, hemoglobin; Plt, platelet; Fib, fibrinogen; T-SAT, transferrin saturation; CRP, C-reactive protein.

**Table 2:** Ultrasound findings and parameters at catheter removal.

	All (n = 37)	Thrombus Group (n = 11)	Non-Thrombus Group (n = 26)	*p*-Value
Catheter diameter (12Fr:13Fr)	27:10	8:3	19:7	0.98
Catheter indwelling days (days)	17.0 (14.0 to 26.0)	21.0 (15.5 to 22.0)	16.5 (14.0 to 27.8)	0.67
Thrombosis at catheter removal, n (%)	37 (100.0)	11 (100.0)	26 (100)	
- ^a^ FS type, n (%)	28 (75.7)	7 (63.6)	21 (80.8)	<0.01
- Mixed thrombus and FS type, n (%)	5 (13.5)	0 (0)	5 (19.2)	
- CTO type, n (%)	4 (10.8)	4 (36.4)	0 (0)	
Enlarged lymph nodes, n (%)	8 (21.6)	5 (45.5)	3 (11.5)	0.04
Symptoms, n (%)	12 (32.4)	2 (18.2)	10 (38.5)	0.23
Bacteremia, (%)	2 (5.4)	0 (0)	2 (7.7)	0.34
Phlebitis, n (%)	1 (2.7)	1 (9.1)	0 (0)	0.12
WBC (/μL)	5700 (4300 to 8400)	7300 (5600 to 8550)	5050 (4000 to 7875)	0.17
Hb (g/dL)	8.9 (8.2 to 11.0)	8.5 (7.8 to 11.0)	9.5 (8.3 to 11.0)	0.55
Plt (×10^5^/μL)	15.9 (10.1 to 21.8)	17.8 (12.7 to 25.4)	13.2 (7.6 to 21.4)	0.19
Fib (mg/dL)	381 (299 to 456)	381 (257 to 504)	377 (316 to 455)	1.00
D-dimer (μg/mL)	8.5 (4.8 to 13.7)	5.8 (3.3 to 8.9)	9.3 (5.5 to 15.7)	0.08
CRP (mg/dL)	0.8 (0.2 to 4.5)	0.46 (0.14 to 4.82)	0.8 (0.2 to 3.6)	0.93

^a^ FS, fibrin sheath; CTO, Complete thrombotic occlusion; Hb, hemoglobin; Plt, platelet; Fib, fibrinogen; CRP, C-reactive protein.

**Table 3:** Change rate of parameters during the catheter indwelling period.

	All (n = 37)	Thrombus Group (n = 11)	Non-Thrombus Group (n = 26)	*p*-Value
WBC change rate (%)	0 (−24.6 to 28.6)	2.2 (−12.9 to 32.4)	−3.5 (−36.1 to 18.1)	0.23
^a^ Hb change rate (%)	0.8 (−14.5 to 6.0)	−9.4 (−25.4 to 2.9)	3.0 (−10.6 to 9.9)	0.04
Plt change rate (%)	5.6 (−16.5 to 34.7)	5.4 (−15.9 to 21.9)	6.8 (−17.4 to 37.4)	0.83
Fib change rate (%)	−5.1 (−19.6 to 17.5)	−7.7 (−22.9 to 17.8)	−1.3 (−20.4 to 18.0)	0.74
D-dimer change rate (%)	74.3 (−2.6 to 286.1)	58.9 (−58.8 to 290.9)	76.1 (6.9 to 291.3)	0.61
CRP change rate (%)	−33.3 (−69.4 to 163.9)	−34.0 (−73.5 to 285.8)	−27.7 (−69.4 to 145.8)	0.91

^a^ Hb, hemoglobin; Plt, platelet; Fib, fibrinogen; CRP, C-reactive protein.

**Table 4:** Odds ratios for AV access dysfunction (a), enlarged lymph nodes (b), and Hb change rate (c) with residual IJV thrombosis one month after dialysis catheter removal.

	Covariates	Odds Ratio (95% CI)	*p*-Value
(a) ^a^ AV access dysfunction			
1	Crude	6.39 (1.18–34.62)	0.03
2	1 + age	6.34 (1.11–36.25)	0.04
3	2 + sex	6.21 (1.09–35.49)	0.04
4	3 + DM	8.06 (1.16–56.28)	0.04
(b) Enlarged lymph nodes			
1	Crude	6.39 (1.18–34.62)	0.03
2	1 + age	6.74 (1.13–40.08)	0.04
3	2 + sex	9.45 (1.34–66.65)	0.02
4	3 + DM	8.75 (1.22–62.90)	0.03
(c) Hb change rate			
1	Crude	0.95 (0.90–1.00)	0.04
2	1 + age	0.96 (0.90–1.02)	0.18
3	2 + sex	0.95 (0.90–1.00)	0.07
4	3 + DM	0.95 (0.90–1.01)	0.10

^a^ AV, arteriovenous; DM, diabetes mellitus; Hb, hemoglobin.

## Data Availability

The raw data supporting the conclusions of this article will be made available by the authors upon request.

## Supplementary Materials

The following supporting information can be downloaded at https://www.mdpi.com/article/10.3390/jcm13247579/s1, Figure S1: Flow chart of the cohort.
