# Supplementary figures and images for: Risk Factors for Internal Jugular Vein Thrombosis 1 Month After Non-Cuffed Hemodialysis Catheter Removal
Source: J Clin Med. 2024 Dec 13;13(24):7579. doi: 10.3390/jcm13247579 (PMC11679927; doi:10.3390/jcm13247579)

Figure S1. Flow chart of the cohort.

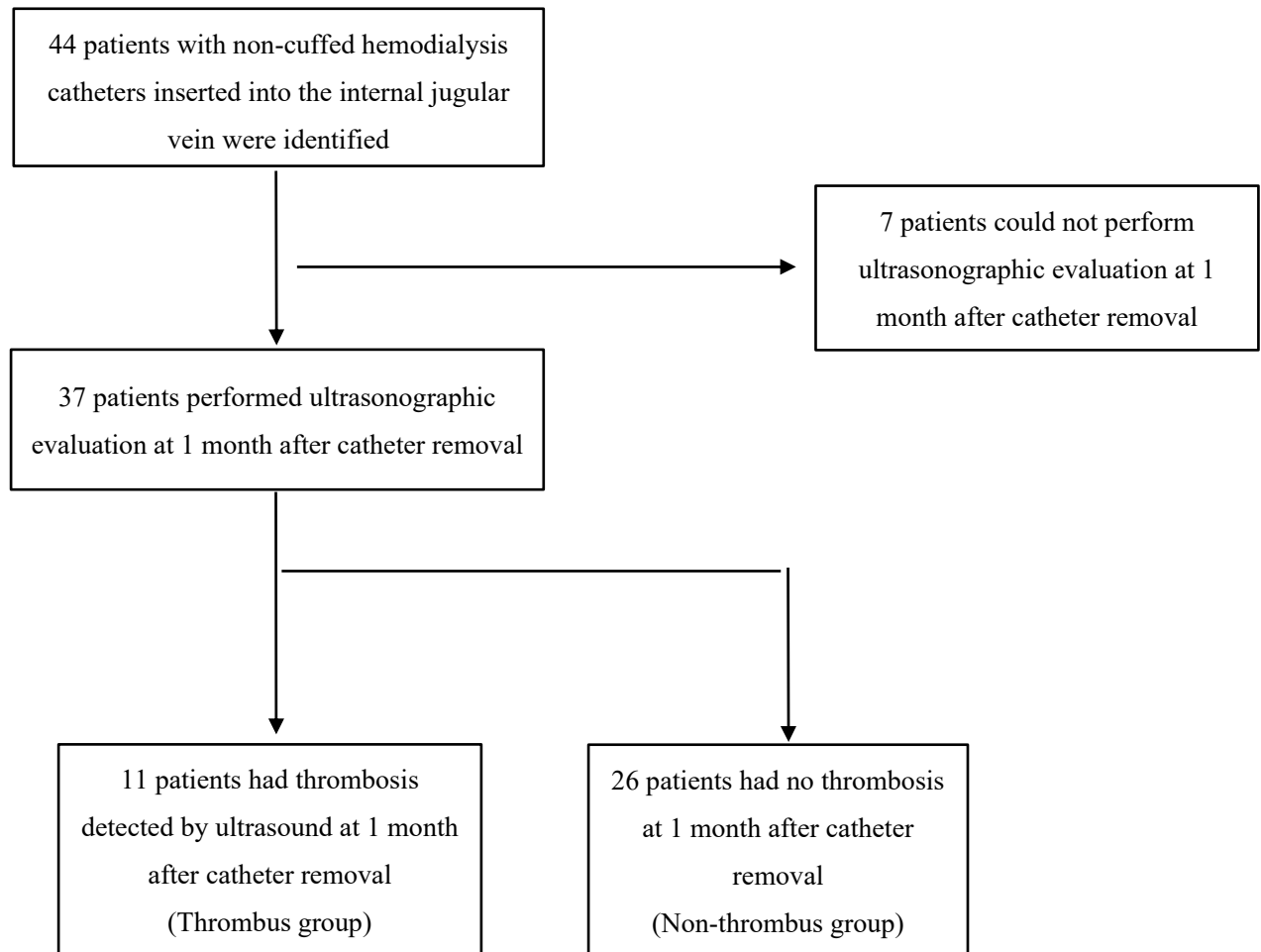

Supplement: Supplementary file 1 [file jcm-13-07579-s001.zip › jcm-3344674-supplementary.pdf]
